# Comprehensive Genome Identification of the *PHD* Gene Family in *Panax ginseng* and Expression Analysis of *PgPHD* Genes in Response to MeJA

**DOI:** 10.3390/biology15151251

**Published:** 2026-07-30

**Authors:** Zifan Xu, Dazhun Guan, Meiyan Fan, Yang Li, Kexin Zhang, Yu Zhang, Kangyu Wang, Meiping Zhang, Mingzhu Zhao, Yi Wang

**Affiliations:** 1 College of Life Science, Jilin Agricultural University, Changchun 130118, China; xuzifan0228@163.com (Z.X.); guandazhun123456@163.com (D.G.); fmy18247587123@163.com (M.F.); ly2664342375@163.com (Y.L.); zkx12302023@163.com (K.Z.); yuzi_zhang0913@163.com (Y.Z.); kangyu.wang@jlau.edu.cn (K.W.); meiping.zhang@jlau.edu.cn (M.Z.); 2 Jilin Engineering Research Center Ginseng Genetic Resources Development and Utilization, Jilin Agricultural University, Changchun 130118, China

**Keywords:** *Panax ginseng*, *PHD* gene family, plant homeodomain finger proteins, Methyl jasmonate, gene expression patterns

## Abstract

Plant homeodomain (PHD) finger proteins are important histone modification readers that play broad roles in the epigenetic regulation of plant development and stress responses. However, the *PHD* finger gene family has not been systematically identified in *Panax ginseng*, and its association with ginsenoside biosynthesis remains unclear. In this study, we identified 63 *PgPHD* genes, corresponding to 163 transcripts, from the *P. ginseng* transcriptome using a combination of HMMER-based domain and BLAST-based homology searches (BLAST+ 2.16.0). Phylogenetic analysis classified these genes into 15 groups. Promoter cis-element prediction, co-expression network construction, and methyl jasmonate (MeJA) induction experiments were further performed to explore the possible regulatory associations of *PgPHD* genes. The *PgPHD* genes were distributed across all 24 chromosomes, and 18 segmentally duplicated gene pairs suggested that whole-genome duplication drove the expansion of this gene family. A total of 308 MeJA-responsive cis-elements were identified in the promoter regions, making them one of the most prevalent functional categories. Co-expression network analysis based on 42 landraces identified five candidate genes significantly associated with key enzyme genes involved in ginsenoside biosynthesis, among which *PgPHD31-09* showed a significant expression correlation with nine key enzyme genes. qRT-PCR analysis showed that *PgPHD31-09* was significantly upregulated in response to MeJA treatment, and its expression pattern was similar to that of several key enzyme-encoding genes related to ginsenoside biosynthesis. This study provides the first systematic analysis of the evolutionary and expression characteristics of the *PHD* gene family in *P. ginseng*, identifying *PgPHD31-09* as a candidate gene potentially associated with MeJA responsiveness and ginsenoside biosynthesis.

## Simple Summary

The *PHD* gene family in ginseng was identified and systematically analyzed, many *PgPHD* genes showed regions in their DNA that respond to the plant hormone methyl jasmonate. Using a combination of computational and experimental approaches, several *PgPHD* genes were found to be linked to genes encoding enzymes that make ginsenosides. Specifically, *PgPHD31-09* increases following MeJA treatment and correlated strongly with the expression of nine key ginsenoside enzyme genes. This suggests that *PgPHD31-09* is a candidate gene associated with MeJA-responsive expression patterns and ginsenoside biosynthesis-related pathways.

## 1. Introduction

Plant homeodomain (PHD) fingers are zinc-finger domains that are widely distributed in eukaryotes and consist of approximately 50–80 amino acid residues. They coordinate two zinc ions through a conserved Cys_4_-His-Cys_3_ motif, forming a cross-brace topology [1]. Within epigenetic regulatory networks, PHD domains primarily function as readers of histone modifications by specifically recognizing distinct methylation states on the histone H3 tail, such as H3K4me2/3, H3K9me3, or unmodified H3K4, thereby converting histone code information into downstream transcriptional regulatory signals [2,3,4]. In addition to the core PHD domain, family members often harbor auxiliary domains, such as SET, BAH, PWWP, and Alfin, and the differential combinations of these domains confer highly diverse biological functions to PHD proteins [5,6].

In plants, PHD finger proteins have been shown to be involved in flowering time regulation [7,8], pollen and tapetum development [9], vernalization response [10], seed germination [11], and abiotic stress responses, such as salinity and drought [12,13]. With the accumulation of genomic data, this family has been identified at the genome-wide level in multiple species, including *Arabidopsis thaliana* [14], *Oryza sativa* [15], *Zea mays* [16], *Glycine max* [17], *Populus trichocarpa* [18], *Brassica rapa* [19], and *Triticum aestivum* [20], with family sizes ranging from 44 members in Arabidopsis to 244 in wheat. Whole-genome duplication (WGD) events are considered important factors driving variations in family size [15,20]. However, these studies have mainly focused on food crops and model plants, and the composition and functional associations of this family in medicinal plants have not yet been systematically reported.

*Panax ginseng* C.A. Meyer is a perennial herbaceous plant of the Araliaceae family, and its principal pharmacologically active constituents are dammarane-type triterpene saponins, known as ginsenosides. Ginsenoside biosynthesis involves multiple enzymatic reactions and can be broadly divided into three stages: upstream synthesis of isoprenoid precursors (IPP/DMAPP), midstream elongation and cyclization of the carbon skeleton, and downstream oxidative modification and glycosylation of the aglycone [21]. Key enzymes in this pathway include FPS, SS, SE, DDS/β-AS, cytochrome P450 oxidases, and UGT glycosyltransferases [22]. Methyl jasmonate (MeJA) is one of the most effective exogenous induction signals in this pathway [23,24]. In recent years, several transcription factor and protein kinase families, such as WRKY, bHLH, and WNK, have been reported to participate in the regulation of ginsenoside biosynthesis [25,26,27]. However, these studies have exclusively focused on transcription factors and signal kinases, and the involvement of histone modification reader proteins in MeJA signal-mediated regulation of saponin biosynthesis remains unclear.

Based on the above background, the present study aimed to systematically identify the *PHD* gene family in *P. ginseng* and characterize their phylogenetic relationships, gene structures, conserved domains, chromosomal distribution, and expression profiles across multiple tissues, developmental stages, and landraces. Furthermore, by integrating promoter cis-element prediction, co-expression network analysis, and MeJA-elicited qRT-PCR validation, we screened for candidate *PgPHD* genes potentially associated with MeJA responsiveness and ginsenoside biosynthesis. This study provides candidate genes and foundational data for future investigations into the biological functions of *PgPHD* genes and their molecular mechanisms in the epigenetic regulation of ginsenoside metabolism.

## 2. Materials and Methods

### 2.1. Identification and Nomenclature of PgPHD Genes

In this study, a dual-channel identification strategy integrating HMM-based domain and sequence homology searches was employed to comprehensively identify members of the *PHD* gene family in *P. ginseng*. First, the hidden Markov model corresponding to the PHD domain (PF00628) was retrieved from the Pfam database (http://pfam.xfam.org/ accessed on 1 December 2025) and used to perform a global search of the *P. ginseng* transcriptome database using HMMER 3.0 (hmmsearch) [28], with the E-value threshold set at 1 × 10^−5^ to ensure search sensitivity, yielding a preliminary set of transcripts containing the PHD domain. Second, functionally characterized plant *PHD* gene sequences were retrieved from GenBank and used as query templates for BLASTN (BLAST+ 2.16.0) homology searches against the same transcriptome database, with an E-value threshold of 1 × 10^−5^, to obtain another set of candidate transcripts. The sequences obtained from the two approaches were merged, and redundant sequences were removed from the merged sequences. To eliminate false positives, spurious sequences were filtered out using iTAK (http://itak.feilab.net/cgi-bin/itak/index.cgi accessed on 8 December 2025), and all candidate sequences were further cross-verified for conserved domains using the two online platforms SMART (https://smart.embl.de/ accessed on 10 December 2025) and NCBI CD-Search (https://www.ncbi.nlm.nih.gov/Structure/cdd/wrpsb.cgi accessed on 11 December 2025). Sequences lacking complete PHD domains were removed. In addition, transcripts without complete open reading frames were excluded. The PgPHD family members were obtained using the above screening procedures for subsequent analyses.

### 2.2. Phylogenetic Analysis of the PgPHD Family

To elucidate the subfamily differentiation and functional attribution of PgPHD proteins, we constructed a phylogenetic tree primarily for functional classification based on well-characterized model species. *A. thaliana* and *O. sativa* were included as reference species for comparative analyses. Rice PHD protein sequences were obtained from the Rice Genome Annotation Project database (http://rice.plantbiology.msu.edu/ accessed on 16 December 2025), and Arabidopsis PHD protein sequences were retrieved from TAIR (https://www.arabidopsis.org/ accessed on 16 December 2025). Based on published classification studies, one representative PHD protein was selected from each known subfamily of *A. thaliana* and *O. sativa*, prioritizing functionally characterized members, or those with a complete PHD domain when characterized members were unavailable. Multiple sequence alignment was performed using MAFFT v7 with the L-INS-i strategy [29], and the resulting alignment was trimmed using TrimAl with the -automated1 parameter and used for tree construction [30]. Phylogenetic relationships were inferred using the maximum likelihood (ML) method in MEGA11 (https://www.megasoftware.net/ accessed on 18 December 2025) [31]. The optimal amino acid substitution model was selected using the built-in Model Test function in MEGA11 based on the Bayesian Information Criterion (BIC) and determined to be rtREV+G+F. Rate heterogeneity among sites was approximated using a gamma distribution with five discrete categories, and the statistical support for each branch node was evaluated using 1000 bootstrap replicates. The resulting phylogenetic tree was visualized and annotated using iTOL (https://itol.embl.de/ accessed on 20 December 2025) [32].

### 2.3. Motif Composition and Domain Characteristics of PgPHD Genes

To analyze the conserved motif composition and domain characteristics of PgPHD proteins, the PgPHD protein sequences were subjected to conserved motif prediction using the MEME online platform (https://meme-suite.org/meme/ accessed on 25 December 2025) [33]. The parameters were set as follows: the maximum number of motifs was 10, the search mode was set to “Zero or One Occurrence Per Sequence” (ZOOPS), and all other parameters were kept at their default values. Subsequently, the PgPHD protein sequences were submitted to the NCBI Batch CD-Search tool (https://www.ncbi.nlm.nih.gov/Structure/bwrpsb/bwrpsb.cgi accessed on 27 December 2025) for analysis of their domain composition, and the results were visualized using TBtools II software [34].

### 2.4. Chromosomal Distribution and Collinearity Characterization Analysis of PgPHD Genes

To determine the physical distribution and duplication mechanisms of the *PgPHD* gene family in the *P. ginseng* genome, PgPHD transcript sequences were first aligned to the *P. ginseng* genome sequences using BLASTN (BLAST+ 2.16.0). Chromosomal localization was performed at the transcript level. Based on gene localization and chromosomal annotation information, transcripts showing sequence identity ≥ 95% and alignment scores ≥ 200 were screened, and their physical positions on the 24 chromosomes of *P. ginseng* were visualized using the MG2C online tool (http://mg2c.iask.in/mg2c_v2.1 accessed on 30 December 2025). Intraspecific collinearity analysis was performed on these highly homologous transcripts to identify potential gene duplication events, and a Circos plot was generated using the circlize package in R v4.5.3 [35] to visualize the distribution patterns of collinear gene pairs across chromosomes.

### 2.5. Transcriptional Expression Profiling of the PgPHD Family

To characterize the expression patterns of the *PgPHD* gene family in *P. ginseng*, we systematically extracted expression data for PgPHD genes from the Jilin Ginseng Transcriptome Database [36]. The analyzed samples included roots from plants of four different root ages (5, 12, 18, and 25 years), 14 tissues from four-year-old plants (leaf peduncle, leaflet pedicel, fruit peduncle, fruit flesh, main root epidermis, seed, fruit pedicel, leg roots, main root cortex, rhizome, arm roots, fiber roots, leaf blade, and stem), and roots from 42 four-year-old landraces (designated S1–S42). All samples were collected in late July 2010 in Jilin Province, China. During data analysis, transcripts with zero expression across all samples in a given dataset were first excluded, and those with expression activity were retained for subsequent analyses. Expression profiles were visualized using TBtools II software [34].

### 2.6. Cis-Acting Element Prediction in the Promoter Regions of PgPHD Genes

To explore the molecular regulatory characteristics of the transcriptional regulation of *PgPHD* family members, a systematic prediction of cis-acting elements in their promoter regions was performed in this study. Using the translation start site of each gene as the anchor point, 2-kb upstream sequences were extracted as candidate promoter regions and submitted to the PlantCARE database (https://bioinformatics.psb.ugent.be/webtools/plantcare/html/ accessed on 10 January 2026) [37] for the automated identification of functional cis-acting elements. The identified elements were classified and statistically summarized into four functional categories: hormonal signaling response, light response, environmental stress response, and developmental program regulation. Unannotated entries, core promoter elements that are universally present in most eukaryotic genes and elements unrelated to the research focus were excluded from comparative analysis. Element type abundance and distribution frequencies were organized using TBtools II software [34] and visualized graphically.

### 2.7. Construction of the Intra-Family Co-Expression Network

To explore the correlation relationships among *PgPHD* gene family members at the transcriptional level, a co-expression network was constructed based on the expression data from 42 *P. ginseng* landraces [36]. PgPHD transcripts with detectable expression levels in at least one landrace were first selected, and pairwise Pearson correlation coefficients were calculated using the SPSS software (v26.0). Co-expression analysis between PgPHD genes and key ginsenoside biosynthesis enzyme genes was performed using IBM SPSS Statistics (v26.0). The expression data (TPM-normalized) were directly imported. Bivariate Pearson correlations with two-tailed significance tests were computed. Correlation pairs with |r| ≥ 0.8 and *p* < 0.05 were considered significant. The correlation matrix was visualized using the pheatmap package in R. A correlation matrix was constructed using R, and the resulting matrix was imported into BioLayout version 3.4 [38] to convert it into a network topology graph for visualization and interpretation.

### 2.8. Co-Expression Network Analysis of PgPHD Genes with Key Ginsenoside Biosynthetic Enzyme Genes

To screen candidate *PgPHD* genes associated with ginsenoside biosynthesis, candidate *PgPHD* members were selected from two dimensions for a correlation analysis with ginsenoside pathway enzyme genes. First, 15 transcripts that maintained high connectivity under a stringent threshold of *p* ≤ 1.0 × 10^−5^ were extracted from the intra-family co-expression network. Second, the top five transcripts ranked by the number of MeJA-responsive elements were selected from the promoter element analysis (*PgPHD30-01*, *PgPHD30-02*, *PgPHD54-04*, *PgPHD54-02*, and *PgPHD54-05*). Pairwise Pearson correlation coefficients were calculated between the above two groups of 20 candidate transcripts in total and 20 core enzyme genes of the ginsenoside biosynthetic pathway, and a heatmap was generated using R to visualize the correlation strength. On this basis, a co-expression network comprising all 20 candidate PgPHD transcripts and the complete set of core enzyme genes (*CAS_11*, *CAS_13*, *CAS_14*, *CAS_17*, *CAS_23*, *SS_1*, *SE2_1*, *SE2_2*, *SE2_4*, *DS_1*, *DS_3*, *FPS_22*, *AS_14*, *AS_3*, *AS_6*, *AS_7*, *CYP716A53v2_1* [PgCYP137], *CYP716A52v2_3* [PgCYP311], *CYP716A47_1* [PgCYP339], and *UGT71A27_2*) [21] was constructed, and the network topology was analyzed under different *p*-value thresholds.

### 2.9. Transcriptional Response Detection of Candidate PgPHD Genes Under MeJA Induction

Methyl jasmonate (MeJA) is widely demonstrated as a key signaling molecule that induces ginsenoside biosynthesis [39]. To verify the transcriptional response characteristics of candidate *PgPHD* genes to this signal, a hormone treatment experiment was conducted using ginseng adventitious roots induction system. First, the ginseng adventitious root culture system was established by transferring approximately 1.0 g of uniformly growing and contamination-free adventitious roots derived from an established aseptic culture was transferred into 250 mL Erlenmeyer flasks containing 150 mL of Murashige and Skoog (MS) liquid medium. The culture medium was sterilized by autoclaving at 121 °C for 20 min. All culture vessels and instruments were sterilized before use, and all transfers were performed aseptically in a laminar-flow cabinet. Because the adventitious roots were obtained from an established aseptic in vitro culture, no additional surface sterilization was performed before transfer. The cultures were maintained at 22 °C and 110 rpm under constant temperature shaking conditions for 21 d until a stable proliferation phase was reached. Induction treatment was initiated on day 22, and MeJA was added to the culture medium at a final concentration of 200 μM. The final concentration of 200 μM MeJA was selected based on our established laboratory experimental system used in previous studies [27]. Sample collection covered a complete time window of 0–120 h post-induction. Samples were collected at 12 time points (0, 6, 12, 24, 36, 48, 60, 72, 84, 96, 108, and 120 h post-treatment), with three independent biological replicates at each time point. The quantitative detection procedure at the transcriptional level was as follows. Total RNA was isolated and purified using TRIzol reagent, and after quality verification, cDNA templates were synthesized via reverse transcription. qRT-PCR was performed using the UltraSYBR OneStep RT-qPCR Kit (Low ROX, CWBIO, Beijing, China) on an Applied Biosystems 7500 real-time fluorescence quantitative PCR system for amplification and signal acquisition. The qRT-PCR conditions were set as follows: 95 °C for 10 min, 95 °C for 15 s, 60 °C for 60 s, and 40 cycles. The detection targets included five candidate *PgPHD* genes (*PgPHD31-09*, *PgPHD37-05*, *PgPHD48-05*, *PgPHD54-04*, and *PgPHD55-01*) and five key ginsenoside pathway enzyme genes (*PgCYP137*, *PgDS_3*, *PgUGT71A27_2*, *PgSE2_4*, and *PgFPS_22*). In this study, the *Actin 1* gene was used as the reference gene, and all the primers used and their sequences are listed in Table 1. Three technical replicates were performed for each sample. Relative expression levels were calculated using the 2^−ΔΔCt^ method, with the 0 h time point as the calibrator. The formula used was: 2^−[(Ct-target,time X−Ct-ref,time X)−(Ct-target,0h−Ct-ref,0h)]^. One-way ANOVA was then performed directly on these relative expression values to evaluate overall differences among time points, rather than on ΔΔCt values. Data are presented as mean ± SD.

## 3. Results

### 3.1. Identification and Nomenclature of PHD Genes in P. ginseng

Using multiple complementary screening strategies in the *P. ginseng* transcriptome database, we initially identified 247 candidate transcripts containing PHD domains. After filtering spurious sequences using the iTAK program and verifying conserved domains with the NCBI CD-Search and SMART online platforms, 84 transcripts were found to have incomplete domains or lacked valid open reading frames (ORFs). Ultimately, 163 transcripts possessing intact PHD domains and complete ORFs were confirmed, corresponding to 63 genes in the genome (Table S1). Family members were numbered sequentially from *PgPHD01* to *PgPHD63*, and different transcripts derived from the same gene were distinguished by numerical suffixes (e.g., -01, -02). The subsequent analyses of this study were all conducted at the transcriptional level, in order to fully explore the differential information among different transcriptional variants of the genes.

### 3.2. Phylogenetic Analysis of the PgPHD Gene Family

**Figure 1:**
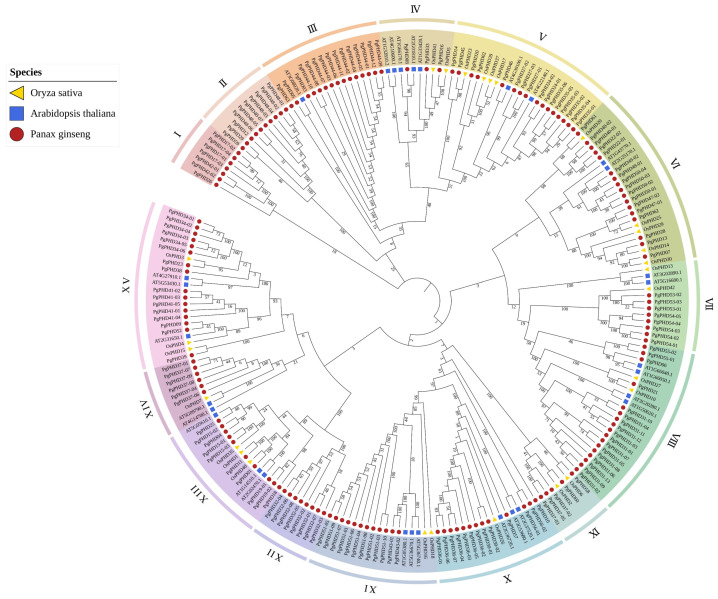
Phylogenetic relationships of the *PgPHD* gene family with the reference species. The tree was constructed using the maximum-likelihood (ML) method based on PHD protein sequences in *Panax ginseng*, aligned with PHD protein sequences from *Arabidopsis thaliana* (At) and *Oryza sativa* (Os), with 1000 bootstrap replicates. Roman numerals I–XV indicate the 15 groups, and the different branch colors denote different groups.

To elucidate the evolutionary relationships of PHD proteins in *P. ginseng*, a phylogenetic tree was constructed using PHD proteins from *Arabidopsis thaliana* (At) and *Oryza sativa* (Os) as reference species (Figure 1). Based on tree topology, all PHD proteins were classified into 15 groups (Groups I–XV). Groups VI and VIII were the largest, each containing 17 *PgPHD* members and accounting for 10.43% of the total members. Groups V and XV were the next largest, each containing 16 members (9.82%). Group IV had the fewest members, with only three (1.84) members. Notably, only Groups I and II were exclusively composed of PgPHD proteins, whereas all other groups contained PHD proteins that co-clustered with those from the reference species. This suggests that the *P. ginseng PHD* gene family may have undergone lineage-specific expansion and diversification within a broadly conserved framework.

### 3.3. Motif Composition and Domain Characterization of PgPHD Genes

**Figure 2:**
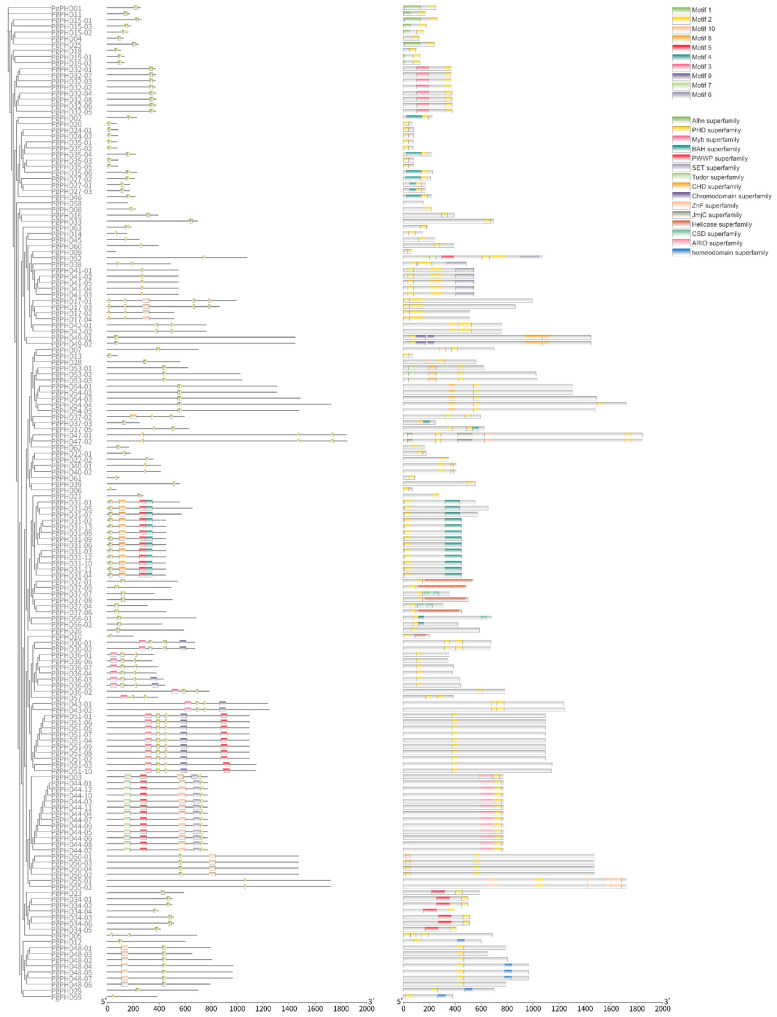
Analysis of conserved motifs and domains of PgPHD proteins. The left panel shows the distribution of conserved motifs, with differently colored boxes representing different conserved motifs (motifs 1–10). The right panel shows the conserved domain composition, with differently colored boxes representing different domains.

Based on the phylogenetic tree constructed from PgPHD protein sequences, combined with MEME motif prediction and conserved domain analysis, the structural features of the family members were systematically characterized. Ten conserved motifs (motifs 1–10) were identified among the 163 *PgPHD* transcripts (Figure 2, Table S2). Motif 2, corresponding to the core PHD domain, was present in one or more copies in all 15 groups. Within each group, the motif composition and arrangement of most members were highly consistent, implying their functional similarity. However, motif combinations differed substantially between groups: Group VIII proteins predominantly contained motifs 1, 2, 4, and 5, whereas Group XI proteins were mainly composed of motifs 2, 5, 6, 7, and 10.

At the domain level (Figure 2), certain domains were restricted to specific groups, suggesting a degree of functional specialization. The Alfin domain is exclusive to Group XIII and plays critical roles in DNA methylation, replication, and transcriptional regulation. The Homeodomain was found only in Group II. As a classic DNA-binding module, it plays a decisive regulatory role in organism development, cell differentiation, and morphogenesis. The overall consistent distribution of motifs and domains within the same group further corroborated the reliability of phylogenetic classification.

### 3.4. Chromosomal Distribution and Collinearity Characterization of PgPHD Genes

**Figure 3:**
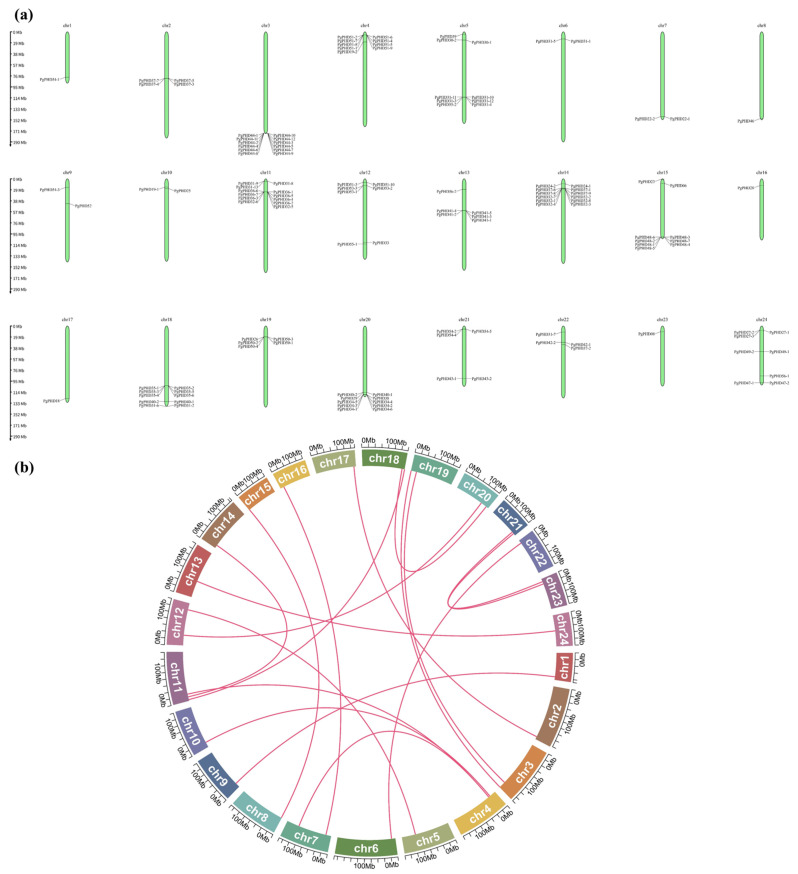
Chromosomal localization and collinearity analysis of the *PgPHD* gene family in ginseng. (**a**) Chromosomal distribution of 135 mapped *PgPHD* genes across the 24 chromosomes of the ginseng genome. The chromosome numbers are indicated at the top, and the scale bar on the left represents megabase (Mb). (**b**) Collinearity analysis of *PgPHD* gene family members within the ginseng genome. Colored blocks represent ginseng chromosomes, and connecting lines indicate segmental duplication gene pairs.

Mapping analysis revealed that 135 of the 163 PgPHD transcripts were successfully anchored to the chromosomes of the *P. ginseng* genome, with *PHD* family members identified on all 24 chromosomes (Figure 3a). The number of *PgPHD* genes per chromosome showed an unequal distribution, ranging from 1 to 12. Chromosomes 3, 11, and 14 harbored the most members (12 each), whereas chromosomes 1, 8, 16, 17, and 23 contained only one member, representing the lowest number across the entire genome. In addition, the distribution of family members on individual chromosomes also showed irregular patterns: genes on chromosome 4 clustered in the upper arm region, those on chromosome 5 were concentrated at both termini, and those on chromosome 24 were relatively evenly dispersed along the entire chromosome. This non-uniform distribution pattern indicates that the positioning of *PgPHD* genes on *P. ginseng* chromosomes is nonrandom. Collinearity analysis identified 18 collinear *PgPHD* gene pairs (Figure 3b). These collinear gene pairs were distributed across different chromosomes, suggesting that segmental duplication is the predominant evolutionary mechanism underlying *PgPHD* gene family expansion.

### 3.5. Transcriptional Expression Profiling of PgPHD Genes

The expression levels of 163 *PgPHD* transcripts were systematically analyzed across roots of four different ages, 14 different tissues of four-year-old *P. ginseng*, and root samples from 42 four-year-old landraces.

**Figure 4:**
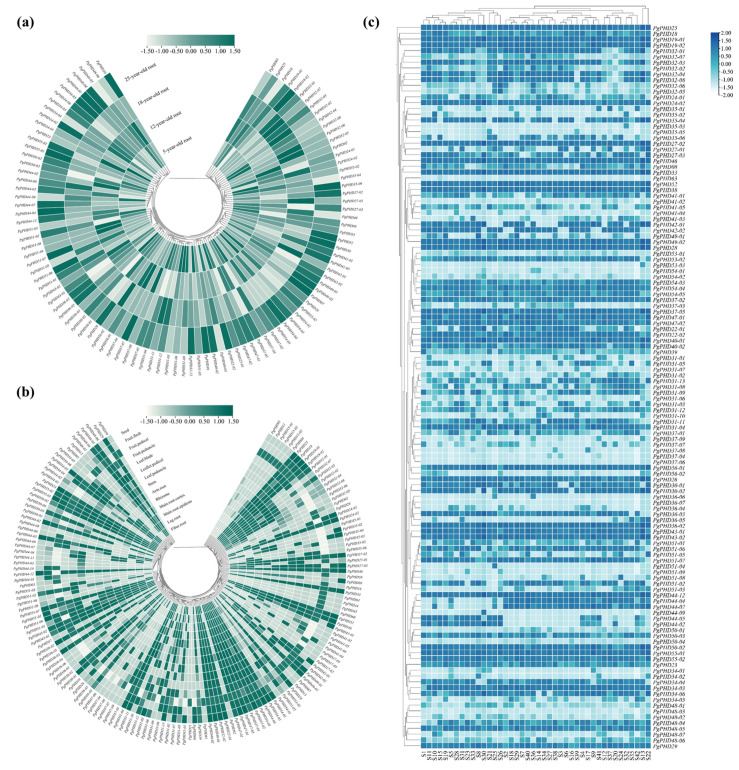
Spatiotemporal expression patterns of *PgPHD* genes in *P. ginseng*. (**a**) Expression of *PgPHD* genes in roots of different ages (5, 12, 18, and 25 years). (**b**) Expression of *PgPHD* genes in 14 different tissues of four-year-old *P. ginseng* plants (leaf peduncle, leaflet pedicel, fruit peduncle, fruit flesh, main root epidermis, seed, fruit pedicel, leg roots, main root cortex, rhizome, arm roots, fiber roots, leaf blade, and stem). (**c**) Expression of *PgPHD* genes in the roots in 42 landraces roots (S1–S42).

In the root development dimension (Figure 4a, Table S3), 84 transcripts (51%) showed no detectable expression across the four age groups. Among the remaining 79 actively expressed transcripts, some reached higher expression at the 18-year-old stage, some were significantly upregulated at the 25-year-old stage, and a few showed higher expression at younger ages, indicating that different *PgPHD* members exhibited divergent expression during root development.

In the tissue expression dimension (Figure 4b, Table S3), nine transcripts were not detected in any of the 14 tissues of four-year-old *P. ginseng* plants. Among the actively expressed transcripts, 40 (24.5%) were expressed in all 14 tissues, and 20 showed tissue-specific expression restricted to a single tissue. Furthermore, *PgPHD31* family members within Group VIII were expressed in multiple tissues, with particularly active expression in root tissues (main root epidermis, main root cortex, leg roots, and fiber roots).

In the landrace expression dimension (Figure 4c, Table S3), analysis of 42 four-year-old *P. ginseng* landraces showed that 38 transcripts showed no expression in any of the landraces. A total of 125 transcripts (76.7%) were expressed in at least one landrace, of which 75 were broadly detected across 30 or more landraces and 23 were detected in fewer than 10 landraces.

### 3.6. Prediction of Cis-Acting Elements in the Promoter Regions of PgPHD Genes

**Figure 5:**
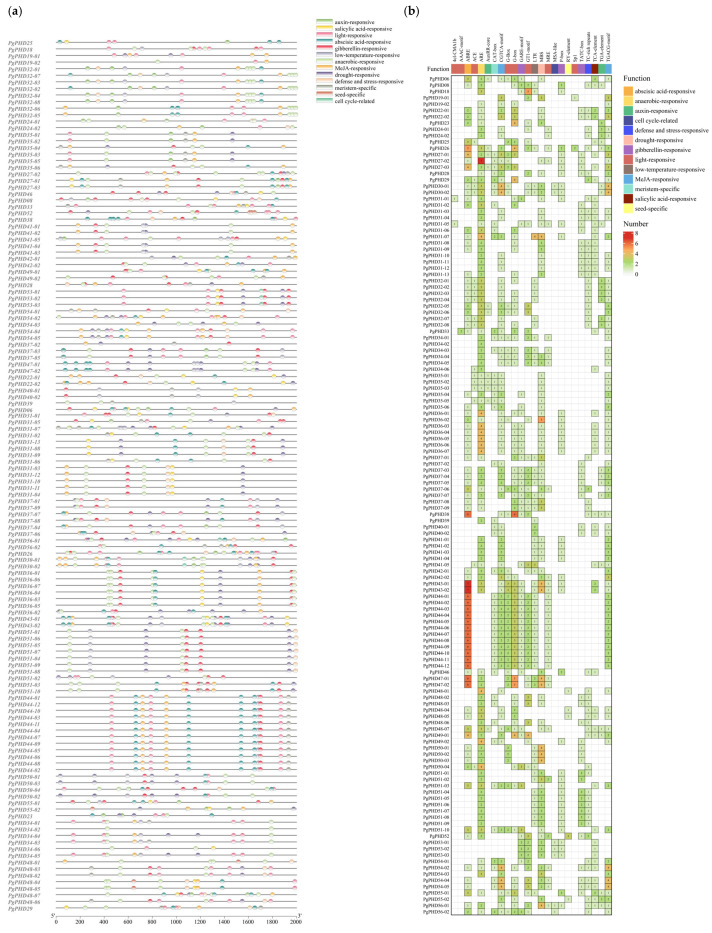
Cis-acting element analysis of the *PgPHD* gene family in *P. ginseng*. (**a**) Prediction of cis-acting elements in the 2-kb upstream promoter regions of *PgPHD* genes. Colored circles represent cis-acting elements with different functions, including hormone-responsive, light-responsive, stress-responsive, and developmental regulation-related elements. (**b**) Heatmap of the quantity and distribution of cis-acting elements in *PgPHD* gene promoters. The color intensity represents the element quantity.

The compositional characteristics of cis-acting elements directly reflect the potential capacity of genes to respond to exogenous stimuli. The prediction results of the cis-acting elements are shown in Figure 5a, and their quantities and distribution patterns are illustrated by a heatmap (Figure 5b). Among the 163 *PgPHD* transcripts, 133 had promoter regions containing at least one identifiable functional element, whereas the remaining 30 had no known elements. In terms of functional classification, the detected cis-elements were divided into four categories: hormone-responsive, light-responsive, stress-responsive, and developmental regulation-related elements. Among them, light-responsive elements were the most numerous; MeJA-responsive elements (TGACG-motif and CGTCA-motif) totaled 308, ranking second among all functional elements; ABA-responsive elements numbered 208; gibberellin-responsive elements numbered 161; and auxin-responsive elements were relatively scarce (46). Among the environmental stress-responsive elements, anaerobic induction elements were the most widely distributed (243), followed by drought-responsive elements (150), low-temperature-responsive elements (70), defense- and stress-related elements (58), and salicylic acid (SA)-responsive elements (58). Developmental regulation elements were relatively limited in number, predominantly comprising meristem-specific elements (54), with cell cycle regulation elements (7) and seed-specific elements (7) sporadically distributed.

Regarding MeJA-responsive element distribution, 94 transcripts carried one or more related elements. *PgPHD30-01*, *PgPHD30-02*, *PgPHD54-04*, *PgPHD54-02*, and *PgPHD54-05* contained eight MeJA-responsive elements, the highest count among all members. The five transcripts enriched in MeJA-responsive elements were selected for further association analysis with key enzyme genes.

### 3.7. Construction of Co-Expression Network Among PgPHD Family Members

**Figure 6:**
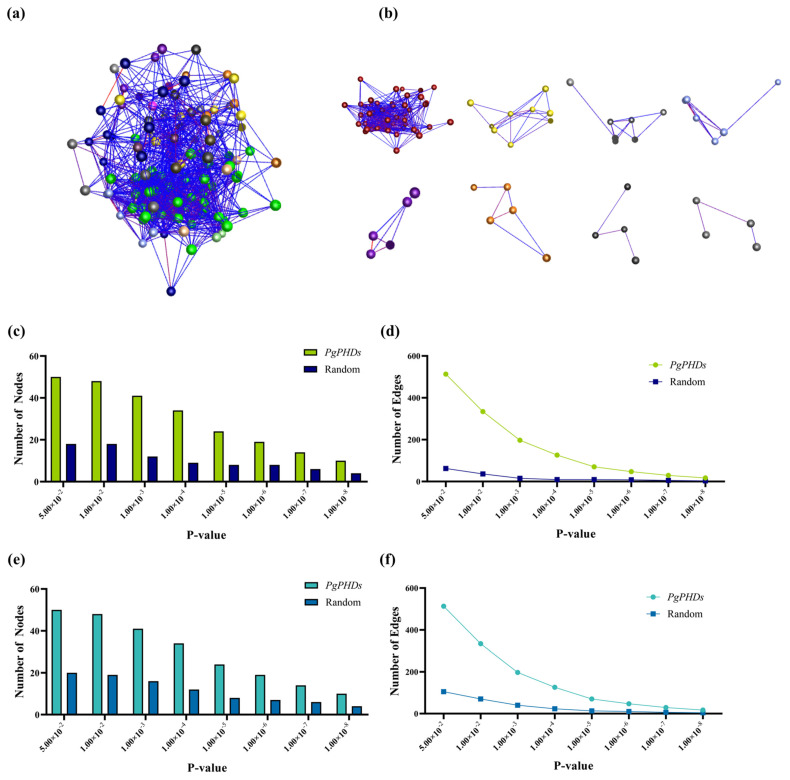
Co-expression network analysis of *PgPHD* gene family members across 42 ginseng landraces. (**a**) Co-expression network of *PgPHD* transcripts constructed under the condition of *p* ≤ 0.05. Nodes represent *PgPHD* transcripts, and edges represent the significant co-expression relationships. (**b**) Under the condition of *p* ≤ 0.05, the 125 transcripts formed eight distinct clusters. (**c**,**d**) Changes in node counts (**c**) and edge counts (**d**) of the network formed at different *p*-value thresholds by randomly selecting another 163 transcripts. Green represents PgPHD transcripts, and blue represents transcripts randomly selected from the *Panax ginseng* transcriptome database as negative controls. (**e**,**f**) Changes in node counts (**e**) and edge counts (**f**) of the network formed at different *p*-value thresholds by randomly selecting two-thirds of the *PgPHD* transcripts. Green represents *PgPHD* transcripts, and blue represents two-thirds of the *PgPHD* transcripts selected as a negative control.

Co-expression network analysis revealed relatively stable correlations among some members of the *PgPHD* family, which were consistent in random control and resampling analyses. Using the 125 transcripts with expression activity across 42 landraces as the analytical subjects, the co-expression network constructed at a threshold of *p* ≤ 5.0 × 10^−2^ contained 50 nodes and 513 edges (Figure 6a), which could be internally divided into eight clustering modules (Figure 6b). When the threshold was gradually tightened to *p* ≤ 1.0 × 10^−8^, the network was reduced to 26 nodes and 26 edges, with the retained gene pairs exhibiting even stronger correlations.

To evaluate whether the above network was significantly better than random expectation, a reference network was constructed using an equal number of non-*PHD* transcripts, as described above. At *p* ≤ 5.0 × 10^−2^, the random network contained only 18 nodes (Figure 6c) and 62 edges (Figure 6d), which further decreased to four nodes and three edges at *p* ≤ 1.0 × 10^−8^, with the connection density far lower than that of the *PgPHD* network, indicating that the co-expression relationships among family members were not attributable to statistical noise. In addition, a network was independently reconstructed using a resampling strategy of randomly selecting two-thirds of transcripts, yielding 20 nodes (Figure 6e) and 105 edges (Figure 6f) at *p* ≤ 5.0 × 10^−2^, and still retaining 12 nodes and 10 edges at *p* ≤ 1.0 × 10^−8^. The network topology was consistent with the full analysis results, further ruling out the dependence of the conclusions on specific sample combinations.

### 3.8. Co-Expression Network Analysis of PgPHD Genes with Key Enzyme Genes of Ginsenoside Biosynthesis

**Figure 7:**
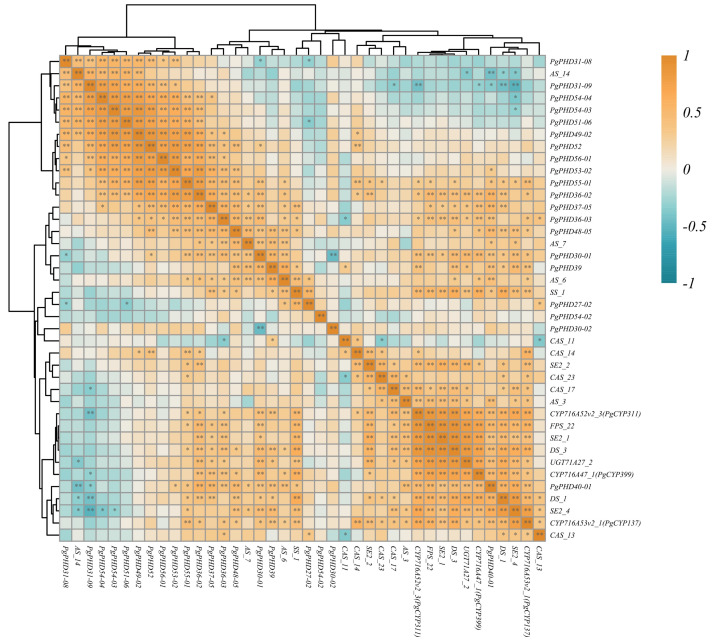
Co-expression correlation coefficient heatmap analysis of 20 *PgPHD* genes with key enzyme genes in ginsenoside biosynthesis. Color gradient represents the Pearson correlation coefficient (orange: positive, blue: negative). Significance was set at |r| ≥ 0.8 and two-tailed * *p* < 0.05, ** *p* < 0.01.

**Figure 8:**
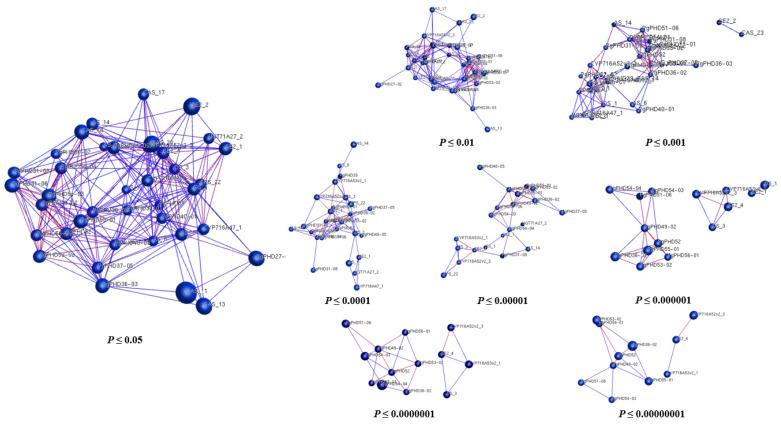
Co-expression network analysis of *PgPHD* genes with key enzyme genes of ginsenoside biosynthesis under different *p*-value conditions (from 0.05 to 0.00000001). Each sub-panel corresponds to a different *p*-value threshold set as the edge filter in BioLayout software: from left to right, *p* ≤ 5.0 × 10^−2^, 1.0 × 10^−3^, 1.0 × 10^−5^, 1.0 × 10^−8^ (two-tailed, based on Spearman correlation, no multiple testing correction). All edges represent correlations with the indicated *p*-value.

Based on the co-expression network analysis within the *PHD* family in *P. ginseng* and the distribution of MeJA-responsive elements in promoters, 20 *PgPHD* transcripts were selected for correlation analysis with key enzyme genes of ginsenoside biosynthesis, and a correlation heatmap was plotted (Figure 7). Changes in network topology were further compared under progressively stringent *p*-value thresholds (5.0 × 10^−2^ to 1.0 × 10^−8^) (Figure 8). Direct connections (straight edges) indicate a statistically significant pairwise Spearman correlation between two nodes, whereas indirect connections (paths through intermediate nodes) imply co-expression relay, potentially reflecting shared regulatory mechanisms or pathway crosstalk. The results showed that as the screening threshold became progressively more stringent, the co-expression associations in the network gradually decreased. Under the threshold of *p* ≤ 1.0 × 10^−5^, five *PgPHD* transcripts retained significant co-expression associations with key enzyme genes, namely *PgPHD31-09*, *PgPHD37-05*, *PgPHD48-05*, *PgPHD54-04*, and *PgPHD55-01*. Among them, *PgPHD31-09* showed significant correlations with nine key enzyme genes (*UGT71A27_2*, *SE2_4*, *SE2_1*, *FPS_22*, *DS_3*, *DS_1*, *AS_14*, *PgCYP137*, and *PgCYP311*), with the number of correlated genes exceeding that of the other four candidate PgPHD transcripts. Based on the above results, these five PgPHD transcripts were selected as detection targets for MeJA induction experiments, with *PgPHD31-09* as the primary focus of subsequent research.

### 3.9. Transcriptional Responses of Candidate PgPHD Genes Under MeJA Treatment

**Figure 9:**
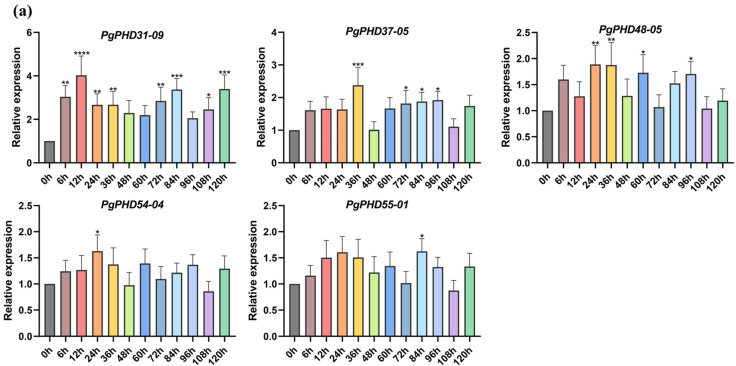

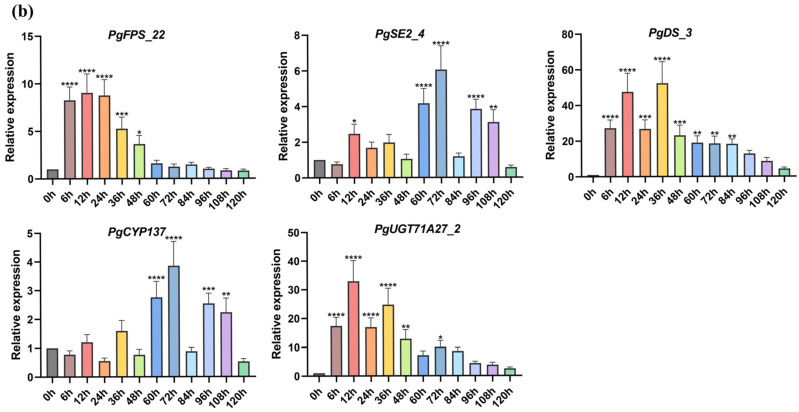
Expression profiles of five candidate *PgPHD* genes and five key ginsenoside biosynthetic enzyme genes in ginseng adventitious roots after MeJA treatment. Data are presented as mean ± standard deviation (SD). The expression levels of the five candidate *PgPHD* genes and five key ginsenoside biosynthetic enzyme genes at different time points (0–120 h) after MeJA treatment were determined using qRT-PCR. Relative expression levels were normalized to those at 0 h as the control (set as 1). (**a**) Expression analysis of candidate *PgPHD* genes (*PgPHD31-09*, *PgPHD37-05*, *PgPHD48-05*, *PgPHD54-04*, and *PgPHD55-01*). (**b**) Expression analysis of key ginsenoside biosynthetic enzyme genes (*PgDS_3*, *PgUGT71A27_2*, *PgFPS_22*, *PgSE2_4*, and *PgCYP137*). Values are the means of three biological replicates. Asterisks indicate significant differences relative to the control (0 h), * *p* ≤ 0.05, ** *p* ≤ 0.01, *** *p* ≤ 0.001, **** *p* ≤ 0.0001.

The relative expression levels of five candidate *PgPHD* genes (*PgPHD31-09*, *PgPHD37-05*, *PgPHD48-05*, *PgPHD54-04*, and *PgPHD55-01*) and five key enzyme genes (*PgCYP137*, *PgDS_3*, *PgSE2_4*, *PgFPS_22*, and *PgUGT71A27_2*) in ginseng adventitious roots were measured at different time points after MeJA treatment. The qRT-PCR results showed that the five candidate *PgPHD* genes (Figure 9a) and the five key ginsenoside biosynthetic enzyme genes (Figure 9b) exhibited distinct response patterns following MeJA treatment.

Overall, the five candidate genes displayed phasic expression changes at approximately 48 h, with some genes showing upregulated expression in both the early and late phases of treatment. *PgPHD31-09* was significantly upregulated as early as 6 h, reaching an initial peak at 12 h, followed by a fluctuating decrease. During the 60–84 h and 96–120 h phases, this gene exhibited two additional expression rebounds, and its expression remained above the control level for the entire 120-h treatment period. In contrast, the expression trends of *PgPHD54-04* and *PgPHD55-01* were similar to those of *PgPHD31-09*, but the first peak was delayed to 24 h. *PgPHD37-05* showed more gradual changes, reaching its peak at 36 h after MeJA treatment. *PgPHD48-05* was significantly upregulated at 24 h in the early phase and at 96 h in the late phase. Compared with the other four candidate genes, *PgPHD31-09* exhibited the most pronounced induction at multiple time points. Based on these results and the co-expression analysis, *PgPHD31-09* was selected as the key candidate gene for subsequent investigations.

The key ginsenoside biosynthetic enzyme genes also showed phasic expression changes at approximately 48 h. *UGT71A27_2*, *DS_3*, and *FPS_22* were predominantly upregulated before 48 h, whereas *SE2_4* and *PgCYP137* were mainly upregulated after 48 h.

## 4. Discussion

Given the critical role of PHD finger proteins in epigenetic regulation and the significant regulatory effect of the MeJA signaling pathway on ginsenoside biosynthesis, this study systematically identified the PHD gene family in *P. ginseng*. In total, the *PgPHD* family comprised 63 genes (corresponding to 163 transcripts), which was larger than the corresponding families in *A. thaliana* (44 genes [14]) and *O. sativa* (59 genes [15]) but smaller than those in *G. max* (95 genes [17]) and *T. aestivum* (244 genes [20]). This relatively large family size is closely related to the two rounds of whole-genome duplication that occurred during ginseng evolution (Pgβ, ~28 million years ago (MYA); Pgα, ~2.2 MYA) [40]. Chromosomal mapping further revealed that *PgPHD* genes were unevenly distributed across the 24 chromosomes, with members of certain subgroups clustered in specific chromosomal regions, suggesting a non-random chromosomal localization. Collinearity analysis identified 18 segmentally duplicated gene pairs, which were presumably derived primarily from the aforementioned two rounds of whole-genome duplication events [40]. A similar expansion pattern has also been reported in the *PHD* families of other polyploid species, including *G. max* [17] and *T. aestivum* [20].

Phylogenetic analysis classified PgPHD proteins into 15 groups, consistent with the number reported in rice [15] and maize [16], greater than that in *B. rapa* (five groups [19]), and fewer than that in Arabidopsis (17 groups [14]) and soybean (20 groups [17]). The variation in group numbers reflects distinct polyploidization histories and post-duplication patterns of gene retention, loss, and expression divergence across species [41]. Among the 15 groups, only Groups I and II contained ginseng-specific *PgPHD* members, whereas members of all other groups co-clustered with proteins from reference species, indicating both evolutionary conservation and lineage-specific expansion within this family.

In addition to the core PHD domain, different groups contained Alfin, PWWP, Myb, BAH, SET, and other domains, with domain combinations varying among groups, consistent with previous reports on *PHD* families in maize [16] and soybean [17]. At the motif composition level, the motif composition within each group was highly consistent, whereas inter-group comparisons revealed substantial differences, except for the conserved Motif 2, further supporting the rationality of the phylogenetic classification. Differences in domain combinations are typically associated with functional divergence of proteins. Studies have shown that, in *Arabidopsis*, AL family proteins possess both PHD and Alfin domains, with the PHD domain specifically recognizing H3K4me3/me2 and the Alfin domain mediating interactions with PRC1, which subsequently recruits PRC2 to promote H3K27me3 accumulation, forming an AL-PRC1-PRC2 regulatory network that regulates plant growth and development [42]. Members of this family have also been implicated in abiotic stress responses, including cold, drought, and high salinity. Cross-species comparison revealed that the rice abiotic stress-responsive gene *OsPHD13* [43] clustered with Group VII *PgPHD* members, and the Arabidopsis salt stress regulator *AtAL5* (AT5G05610.1) [12] clustered with Group XIII *PgPHD* members. Moreover, Group XIII *PgPHD* members share the same PHD-Alfin domain combination as *AtAL5*, suggesting that these subgroup members possess functional characteristics similar to those of previously reported stress-responsive PHD. Notably, abiotic stress is closely associated with ginsenoside biosynthesis and accumulation. Islam et al. [44] reported that salt stress can affect the expression of key enzyme genes involved in ginsenoside biosynthesis, thereby regulating saponin accumulation; meanwhile, the jasmonate signaling pathway in plants simultaneously mediates stress defense and terpenoid secondary metabolic regulation [45]. Based on their phylogenetic relationships with previously reported stress-related PHD proteins, it remains to be determined whether these PgPHD members participate in MeJA-related responses and influence saponin accumulation.

Expression profiling of gene families facilitates the elucidation of transcriptional regulatory synergy and expression divergence among family members. In terms of root development, *PgPHD* family members did not change monotonically with growth age but exhibited significant stage-specific and inter-member divergence. Previous studies have demonstrated that ginsenoside content and composition differ significantly among roots of different ages [46]. The stage-dependent expression divergence of *PgPHD* members corresponds to this pattern, suggesting that certain members are associated with metabolic regulation at different developmental stages of the plant. In terms of tissue expression, most members showed tissue-preferential expression in roots, stems, leaves, fruits, and reproductive organs. Group VIII *PHD* members were expressed in multiple tissues, with particularly active expression in root tissues (including the main root epidermis, main root cortex, leg roots, and fiber roots). Combined with subsequent promoter cis-acting element analysis, which revealed that *PgPHD* members within this subfamily commonly harbored MeJA-responsive elements, Group VIII was prioritized for further investigation. In terms of landrace expression, the *PgPHD* family exhibited pronounced inter-landrace expression divergence, with broadly expressed members likely associated with basal epigenetic regulation, whereas landrace-specifically expressed members may be related to inter-landrace differences in growth characteristics and saponin accumulation. In addition, some transcripts were not detected in any landraces, reflecting strong tissue- or environment-specific expression. Notably, *PgPHD25* maintained relatively high expression levels across different growth ages, tissues, and landraces, exhibiting constitutive expression patterns. Based on phylogenetic analysis, *PgPHD25* clustered with the Arabidopsis H3K4me3 reader *AtAL5* [42] into an orthologous clade, and *AtAL5* also exhibited constitutive expression in expression analyses, suggesting its involvement in the regulation of basal chromatin. The diversity of these expression patterns indicates that *PgPHD* gene family members exhibit functional and expression level divergence across tissues, growth ages, and landraces in ginseng.

Analysis of cis-acting elements in the promoter regions can provide important clues for elucidating the transcriptional regulatory mechanisms of genes and their response to hormone signals and environmental factors. The element profiles in the promoter regions of the individual members exhibited considerable variation. MeJA-responsive elements (TGACG-motif/CGTCA-motif) were highly enriched in *PgPHD* gene promoters, accounting for 17.4% of all detected elements, significantly higher than gibberellin-, ABA-, and other hormone-responsive elements. This distribution pattern differs somewhat from those previously reported for the *PHD* gene families of soybean [17], maize [16], and wheat [20], where ABA-responsive elements predominate. However, Liu et al. [47] also observed significant enrichment of MeJA-responsive elements in the pea *PHD* gene family, which is consistent with our findings. Similar phenomena have been reported in multiple gene families in *P. ginseng*, including FAR1/FHY3 [48], NAC [49], and ABC [50], suggesting that the enrichment of MeJA-responsive elements is a generally prevalent feature in the genomic regulatory regions of ginseng. *P. ginseng*, as a perennial understory plant, is chronically exposed to complex biotic stress environments [51], and the jasmonate signaling pathway plays an important role in its defense responses and secondary metabolism regulation. These results indicate that *PgPHD* genes may play a regulatory role in response to MeJA signaling.

Co-expression network analysis revealed a set of closely associated core members within the *PgPHD* family, and the network topology remained stable under progressively stringent *p*-value thresholds. Randomized control networks and resampling validation further excluded the influence of statistical noise and sample bias on the results. Previous studies have shown that strong intra-family co-expression often indicates similar regulatory mechanisms and functional connections [52]. Similar phenomena have been observed in other gene family studies in ginseng. Jiang et al. [48] reported that some members of the FAR1/FHY3 gene family could form closely connected co-expression modules. These results indicate that *PgPHD* family members exhibit certain associations at the transcriptional level, suggesting potential synergistic interactions among some of them. Based on this characteristic, a joint co-expression network was constructed between *PgPHD* genes and 20 key ginsenoside biosynthetic enzyme genes to screen candidate genes functionally associated with ginsenoside biosynthesis. Under progressively stringent *p*-value thresholds, five candidate transcripts significantly correlated with key enzyme genes were identified. Among them, *PgPHD31-09* was directly connected to the key enzyme genes *AS_14* and *SE2_4* and indirectly associated with *UGT71A27_2*, *SE2_1*, *FPS_22*, *DS_3*, *DS_1*, *CYP716A53v2_1* (PgCYP137), and *CYP716A52v2_3* (PgCYP311), covering multiple catalytic steps from precursor synthesis to scaffold modification in the ginsenoside biosynthetic pathway. It had the broadest range of associations among the five candidates and was therefore selected as the primary target for verification in subsequent MeJA induction experiments.

MeJA treatment further supported the functional association between the candidate genes and ginsenoside biosynthesis. *PgFPS_22*, *PgSE2_4*, *PgDS_3*, *PgCYP137*, and *PgUGT71A27_2* are involved in different stages of the ginsenoside biosynthetic pathway [53]. Among them, *PgFPS* is responsible for the synthesis of the upstream precursor FPP [54], *PgSE* catalyzes the formation of the common precursor 2,3-oxidosqualene [55], *PgDS* further catalyzes the cyclization to generate the dammarane-type triterpene skeleton [53], *PgCYP* participates in the oxidative modification of the triterpene skeleton [56], and *PgUGT* catalyzes terminal glycosylation [57], collectively facilitating ginsenoside biosynthesis. MeJA is an effective signaling molecule that induces ginsenoside biosynthesis in ginseng adventitious roots [58]. In this study, the five candidate genes exhibited member-specific expression patterns after MeJA treatment, consistent with the findings of Wu et al. [59], and displayed phasic expression changes, with 48 h as the inflection point. Among them, *PgPHD31-09* showed the most pronounced response, being rapidly induced in the early phase of treatment, with its early expression peak largely coinciding with the induction of the upstream and midstream key enzyme genes *PgFPS_22* and *PgDS_3* in the ginsenoside biosynthetic pathway. In the late phase, its high expression period coincided with the period of enhanced expression of the downstream oxidative modification key enzyme gene *PgCYP137*. Notably, the induction timing of *PgUGT71A27_2* and *PgSE2_4* did not fully correspond to their catalytic order in the ginsenoside biosynthetic pathway, a phenomenon that has also been reported in MeJA-induced ginsenoside biosynthesis studies [60] and in the secondary metabolic pathways of other plants [61]. *PgPHD31-09* exhibited expression trends similar to those of multiple key enzyme genes during certain time windows, suggesting its association with ginsenoside biosynthesis; however, its specific mode of action requires further investigation. In addition, previous studies have shown that some plant *PHD* family genes exhibit differential expression under multiple hormone treatments, including ABA, IAA, GA, and SA [62], suggesting a close association between this protein family and hormone-responsive regulatory processes in plants. By integrating co-expression analysis, tissue expression characteristics, and MeJA induction results, *PgPHD31-09* was identified as a high-priority candidate gene associated with MeJA response and ginsenoside biosynthesis. However, whether it directly participates in pathway regulation remains to be confirmed through functional experiments.

Although this study preliminarily established an association between the *PgPHD* gene family and ginsenoside biosynthesis, the current evidence is mainly based on co-expression correlations and MeJA transcriptional responses, and the direct regulatory roles of candidate genes, such as *PgPHD31-09*, in ginsenoside biosynthesis have not been confirmed at the functional level. Future studies should advance in the following two aspects: first, validate the function of *PgPHD31-09* through genetic transformation strategies, such as overexpression and CRISPR knockout, to determine its effects on ginsenoside content; and second, use ChIP-seq to map *PgPHD31-09* binding sites at key enzyme gene loci in the ginsenoside biosynthetic pathway, combined with dual-luciferase reporter assays to assess its direct regulatory activity on target gene promoters, thereby establishing the causal relationship between *PHD* proteins and the ginsenoside biosynthetic pathway at the epigenetic level.

## 5. Conclusions

In this study, by integrating phylogenetic analysis, collinearity analysis, promoter cis-acting element prediction, multidimensional transcriptome expression profiling, and co-expression network construction, the *PHD* gene family was systematically identified in *P. ginseng* for the first time. A total of 63 *PgPHD* genes corresponding to 163 transcripts were confirmed, and their subfamily classification, gene structure and conserved motif characteristics, chromosomal distribution, and expression patterns were systematically characterized. Based on this, co-expression network analysis was performed to evaluate the association between candidate genes and key enzyme genes in the ginsenoside biosynthetic pathway. *PgPHD31-09* was identified as a candidate gene potentially associated with MeJA responsiveness and ginsenoside biosynthesis in this study. qRT-PCR analysis after MeJA treatment further supported the correlation between *PgPHD31-09* expression, MeJA responsiveness, and ginsenoside biosynthesis-related gene expression. These results provide a candidate-gene resource for further investigating the potential regulatory roles and molecular mechanisms of *PgPHD* genes in ginsenoside biosynthesis.

## Figures and Tables

**Table 1:** Details of gene primers used in this study for fluorescence quantitative PCR.

Number	Primer Name	Primer Sequences	Product Size
1	Action 1-F	TGGCATCACTTTCTACAACG	150 bp
	Action 1-R	TTTGTGTCATCTTCTCCCTGTT	
2	PgPHD31-09-F	AGAGGTGAGTGGCATTGTGG	84 bp
	PgPHD31-09-R	ATTTCTCATGACACGGCCGT	
3	PgPHD37-05-F	TGACGCGAACTTTCCTCTCC	183 bp
	PgPHD37-05-R	CAGGGCAAAGGGGATTGGAT	
4	PgPHD48-05-F	AGGGAAGATCTCGTGGTCGA	130 bp
	PgPHD48-05-R	CGCAATAATCTGGTGCTGCC	
5	PgPHD54-04-F	ATCCATTTGGGGGCAGACTG	119 bp
	PgPHD54-04-R	GTGGAGACTGGTTCCATGGG	
6	PgPHD55-01-F	CAGCAGAAGTTACAGGGGCA	150 bp
	PgPHD55-01R	ACCTGACTGCCCCACTCTAT	
7	FPS_22-F	CTGGATTGCTTTGGTGCACC	166 bp
	FPS_22-R	CTTTTGCTACAGAGGCCGGA	
8	SE2_4-F	TAATCAAAGCATGCGTCGCG	80 bp
	SE2_4-R	TTGGCTGGTGCGCTATACAA	
9	DS_3-F	AGTATCCACTTCCGGCCTCT	150 bp
	DS_3-R	TGAGGATGGTGGATGGGGAT	
10	PgCYP137-F	CGCTGACTTTTGAGTTGGCC	155 bp
	PgCYP137-R	GGCCTTGATCCCACGGTTAA	
11	UGT71A27_2-F	AGGTGCAGAGAGTCAAAGGC	169 bp
	UGT71A27_2-R	CAAGGATCACAGCAGCCTCA	

## Data Availability

The data used for this study are available at Sequence Read Archive (SRA) of National Center for Biotechnology Information (NCBI) of BioProject PRJNA302556. All ginseng samples can be accessed upon request from the corresponding author.

## Supplementary Materials

The following supporting information can be downloaded at: https://www.mdpi.com/article/10.3390/biology15151251/s1, Table S1: Basic information of *PgPHD01*–*PgPHD63* genes in ginseng; Table S2: Characteristics of the conserved motifs identified in PgPHD proteins using MEME; Table S3: The TPM expressions of the *PgPHD01*–*PgPHD63* genes in 42 cultivars roots, 14 tissues, and 4 aged roots in ginseng.
